# Assessment of relevance and actual implementation of person-centeredness in healthcare and social support services for women with unintended pregnancy in Germany (CarePreg): results of expert workshops

**DOI:** 10.1186/s12884-024-06453-8

**Published:** 2024-04-06

**Authors:** Anja Lindig, Stefanie Heger, Jördis Maria Zill

**Affiliations:** 1 Department of Medical Psychology, University Medical Center Hamburg-Eppendorf, Martinistraße 52, 20246 Hamburg, Germany; 2 Center of Health Care Research, University Medical Center Hamburg-Eppendorf, Martinistraße 52, 20246 Hamburg, Germany

**Keywords:** Abortion care, Unintended pregnancy, Person-centered care, Expert workshops, Qualitative content analysis

## Abstract

**Introduction:**

Person-centeredness is a key principle in the German healthcare system. However, access to high-quality care for women with unintended pregnancy is limited due to social stigma and legal restrictions. There is little research on the adoption of person-centeredness in care for women with unintended pregnancy. The aim of this study was to analyze relevance and actual implementation of dimensions of person-centeredness in psycho-social and medical abortion care from the view of abortion care providers.

**Methods:**

Counselors and gynecologist working in psycho-social or medical abortion care participated in one of two digital workshops. Discussions were semi-structured based on the 16 dimensions of an integrative model of person-centeredness, audio-recorded and transcribed verbatim. During qualitative content analysis, deductive categories based on the integrative model of person-centeredness were applied and inductive categories were developed. Additionally, participants rated relevance and actual implementation of the dimensions in an online survey.

**Results:**

The 18 workshop participants most intensively discussed the dimensions “access to care”, “person-centered characteristics of healthcare providers” and “personally tailored information”. Four additional categories on a macro level (“stigmatization of women with unintended pregnancy”, “stigmatization of healthcare providers”, “political and legal aspects” and “corona pandemic”) were identified. Most dimensions were rated as highly relevant but implementation status was described as rather low.

**Conclusions:**

In Germany, high quality person-centered care for women with unintended pregnancy is insufficiently implemented through limited access to information, a lack of abortion care providers, and stigmatization. There is a need for changes in health care structures to enable nationwide person-centered care for women with unintended pregnancy. Those changes include a more easy access to evidence-based information and person-centered abortion care, more education on abortion care for healthcare providers, integration of topics of abortion care in medical schools and promotion of de-stigmatizing actions to enable abortions as part of the general healthcare.

**Supplementary Information:**

The online version contains supplementary material available at 10.1186/s12884-024-06453-8.

## Introduction

Induced abortion is a simple and commonly performed healthcare procedure. Approximately 73 million induced abortions occur globally each year. Six out of 10 (61%) unintended pregnancies and three out of 10 (29%) pregnancies overall result in induced abortion [1]. The legal regulations of abortion rights and the abortion care situation for women with unintended pregnancy (UP) is currently a highly discussed topic in Germany [2–5]. The German Criminal Code defines abortions as illegal under all circumstances (§218 StGB [6]). However, abortions will be unpunished (for the abortion provider as well as the affected women) if 1) no more than 14 weeks have elapsed since the first day of the last menstruation, the pregnant women took part in a mandatory “pregnancy conflict counseling” provided by a certified social support service center at least three days before the abortion procedure and received a certificate for the attendance (so called “counseling provision”), 2) grave impairment to the physical or mental health of the pregnant women has to be averted, 3) the pregnancy resulted from sexual assault or rape (§218 StGB [7]). In Germany, 94.596 abortions were reported for the year 2021, which is 4.5 abortions per 1.000 women [8]. In contrast, 795.500 children were born in 2021 [9]. We do not know, how many of those were unintended pregnancies carried to term. The vast majority of abortions (95.8%) is conducted after a mandatory pregnancy conflict counseing [8, 10]. The German Act on Assistance to Avoid and Cope with conflicts in Pregnancy (SchKG) addresses the provision and administration of counseling for pregnancy conflicts. The objective of pregnancy conflict counseling is safeguarding the well-being of the unborn child, emphasizing transparency in its conduct, and placing responsibility on the women [11]. Specialized pregnancy conflict counseling service has to be recognized by state. Abortions according to counseling provision are not covered by public health insurances but have to be paid by the affected individual. However, in case of low income, individuals can apply for cost coverage by their health insurances.

In 2020, the highest proportion of women having an abortion was between 30 and 34 years old (24.5%) and more than half of the women had already given birth to children [8]. When having an abortion in case of counseling provision, in 42.2% of women, the gestational age was under seven weeks, in 33.6% it was seven to eight weeks and in 21.0% it was nine to eleven weeks [8]. In general, abortions in Germany are carried out by gynaecologists on an outpatient basis. In the year 2021, 81.0% of the procedures occurred in gynaecological practices or surgical centers, with 15.7% being conducted as outpatient procedures in hospitals, and 3.3% as inpatient procedures [10]. Up to now, there is no specific training on abortion procedures and it is rarely integrated in medical school curricula. Thus, it is very often the responsibility of the gynaecologists themselves to learn the relevant skills. Furthermore, single gynaecologists as well as hospitals have the right to decline offering abortion care, as long it is necessary to avert an otherwise unavoidable risk of death or serious damage to the woman's health [11]. The number of practices and hospitals performing abortions has nearly been halved within the past 20 years. It decreased from 2.050 in 2003 to 1.092 facilities while the overall number of performed abortions remained stable. There are regional differences on travel distances between women’s places of residence and abortion practices and clinics. For example, in over a third (38.7%) of women from Rhineland-Palatinate and in more than one-sixth (18.6%) of women from Lower Saxony, the abortion procedures were conducted in a different federal state, primarily in Saarland or Bremen [10]. Overall in Germany, 52.1% of abortions were conducted by vacuum aspiration, 11.4% by curettage and 32.3% were medical abortions using mifepristone [8]. However, there are large regional differences in the abortion methods primarily used [8].

In 2022, the World Health Organization published a revised guideline on safe abortion care, which should be followed independent of varying legally and politically differences between countries [12]. Recommendations of this guideline are in line with the concept of patient-centeredness, or synonymously person-centeredness (PC), which has been defined as key quality criterion in German healthcare [13, 14]. PC describes a relationship between healthcare providers (HCPs) and individuals, affording the primacy of the individual’s preferences, needs and values [15]. Based on a systematic integration of definitions of PC, Scholl et al. developed the integrative model of PC, consisting of 15 dimensions, which can be split into three groups: principles (e.g. person-centered characteristics of healthcare providers, uniqueness of each person), enablers (e.g. access to care, integration of additional healthcare elements) and activities (e.g. personally tailored information, empowerment of the person) [16]. In a recent study on the view of patients on the integrative model of PC, the dimension “patient safety” was added as 16th dimension [17]. Crucial aspects of PC in abortion care include informed decision-making, confidentiality and privacy in healthcare, and access to legal and affordable care services [12]. In the broader context of reproductive healthcare, PC has shown to improve women’s healthcare outcomes [18]. However, due to restrictive healthcare policies and social stigma, the concept of PC has not found much practical relevance in abortion care and was found to be hardly implemented in healthcare for women with UP [19].

With a few exceptions [20, 21], international studies mainly evaluated experiences of women with UP in abortion care by focusing on their general satisfaction with care and not on PC in abortion care [19, 22]. In a review from Doran & Nancarrow (2015) including 38 articles, women seeking an abortion described following barriers to access abortion care: distance to abortion care services and abortion service availability, negative attitudes of staff and costs [23]. This review is also one of the few international studies integrating attitudes and experiences of abortion care providers. They described following barriers in abortion care: moral opposition of HCPs regarding abortion, lack of training, too few physicians offering abortion care, staff harassment, and insufficient hospital resources [23]. A recent study on women’s experiences of abortion care in the Netherlands defined several barriers to abortion care access including taboos within abortion laws and healthcare, limited accessibility for specific marginalized groups, and women facing challenges in openly discussing abortion [24]. Other studies highlight the burden of women, who have to travel longer distances or even cross-country to have an abortion [25, 26].

In Germany, the data availability on experiences of women with UP and HCPs on abortion care is very limited and the quality of German abortion care is not yet analyzed regarding implementation of PC. Furthermore, there is no study on experiences of abortion care providers in social support services and medical abortion care in Germany.

In 2019, the Federal German Health Ministry provided a funding amount of five million Euros for projects focussing on the “psychosocial situation and support needs of women with unwanted pregnancy”. This funding amount was allocated to three research projects, including the project “Assessment of person-centeredness in healthcare and social support services for women with unintended pregnancy (CarePreg)”, which assesses PC in social support services and medical abortion care in Germany. Details on the CarePreg project can be found in a recently published study protocol [27]. The study at hand is part of the CarePreg project and aims to analyze relevance and actual implementation of dimensions of the integrative model of PC in psycho-social and medical abortion care from the view of abortion care providers.

## Methods

### Study design

This study is part of the CarePreg study which comprises three phases. The first of the three phases of the CarePreg project was a pre-phase with the aim to explore experiences of women with UP as well as HCPs with person-centered abortion care in Germany.

The study at hand is a qualitative study presenting the qualitative results of two workshops with abortion care providers. Those workshops comprise elements of focus groups since both are interactive group activities [28]. However, compared to focus groups, the methodology of our workshops was based on structured discussions leaded by a study team member. Additionally, since we expected the term ‘workshop’ to be more accepted by our participants, we decided to uses this term instead of ‘focus groups’. Participants of our workshops, so-called experts, worked as physicians and counselors in psycho-social and medical care for women with UP. Apart from a few exeptions, physicians performing abortions have a specialized education as gynecologists. In the following we therefore use the term gynecologists to refer to physicians providing abortions. In the workshops we discussed relevance and actual implementation of the 16 dimensions of the integrative model of PC in German abortion care [16]. After the workshops, participants rated relevance and actual implementation of the 16 dimensions of the integrative model of PC in German abortion care [16] in a quantitative online survey.

In the following, this manuscript uses the term “women” to describe people, who can become pregnant. This term also includes non-binary individuals and trans men, who can also be affected by an unintended pregnancy.

Data were collected in April 2021. For presenting the qualitative results of the expert workshops, we followed the consolidated criteria for reporting qualitative research checklist (COREQ, [29], see Additional File 1).

### Recruitment

We conducted two expert workshops with gynecologists and counselors, recruited via a convenience sampling approach. Participants were employees of practices and social support services, which are cooperation partners of the CarePreg project. Additionally, all gynecologists who were listed on the official list of abortion providers of the German federal medical association (german: Bundesärztekammer) were contacted and asked for participation in the workshops. Members of the study team (JZ and LR, see List of Acknowledgements) invited participants to take part in the study either personally or via e-mail. Further information on researcher characteristics can be found in Additional file 2.

### Data collection

The workshops were performed online via the platform Webex (Cisco Webex Meeting, Cisco Systems, San Jose’, California, USA). Each workshop consisted of two parts. In the first part of the workshop (duration about 30 min), participants received an introduction on the background of the CarePreg project as well as the concept and assessment methods of PC, and the integrative model of PC including the 16 dimensions [16]. This part of the workshop was moderated by two researchers of the study team (JZ and LR). Additionally, two student assistants (SH and AI, see List of Acknowledgements) were present. This first part of expert workshops was not audio-recorded. For the second part of the workshop (duration about 2 h), participants were split into two groups and assigned to one of two breakout rooms of the online platform. Equal distribution of participants according to their profession was heeded. The subgroups were either moderated by LR or JZ. In each subgroup, one student assistant (SH or AI,) was present to take meeting minutes. The study team planned to discuss eight of the 16 dimensions of the integrative model of PC in each subgroup. Dimensions of the three categories of the integrative model of PC (principles, enables, activities, [16]) were equally distributed over subgroups. Discussions within subgroups followed a semi-structured guideline [28, 30, 31], which was discussed with researcher of the research group working in the field of PC (PH and IS, see List of Acknowledgements). The guideline was not pilot tested. First, the name of the dimension and a definition was presented to participants together with following questions for the discussion: “What significance does this dimension has for your activity?”, “Which aspects do you think are particularly relevant for an unintentionally pregnant woman?”, “How do you see this dimension implemented in care in Germany?”, “Which aspects should be supplemented?”. Additionally, the items of the measure Experienced Patient-Centeredness Questionnaire (EPAT), which evaluates the 16 dimensions of the integrative model of PC, were presented [32]. The second part of the workshop was audio-recorded and transcribed verbatim by our student assistant AI. No workshop was repeated. For their participation, experts received a compensation fee of 100 Euro. However some of the experts refused compensation or wanted it to be donated.

After taking part in the expert workshops, participants rated the 16 dimensions of the integrative model of PC regarding their relevance and actual implementation in German abortion care (see Additional file 2). For each of the 16 dimensions, participants were asked: “How relevant do you consider this dimension for the care of unintentionally pregnant individuals?”, to be answered on a 10-point scale from “not relevant” (1) to “extremely relevant” (10) and “How well implemented do you find this dimension for the care of unintentionally pregnant individuals in Germany?”, to be answered on a 10-point scale from “not implemented at all” (1) to “largely implemented” (10). Via this online survey, we also assessed demographic data of the participants (e.g. age, gender, confession, work background, education).

### Data analysis

We analyzed the four transcripts using qualitative content analysis [30, 33–36]. First, we defined deductive categories according to the 16 dimensions of the integrative model of PC. Two members of the study team (AL and JZ) each coded 50% of all transcripts (transcript of one of the two subgroups per workshop). During the coding process, inductive codes and subcodes were added to the deductive coding scheme. Statements of participants were assigned to the category, which fits best according to the content of the data, independently from the dimension of the model, which was discussed. For example, if participants discussed the dimension “access to care” but a statement of a participant better fits to the category “planning of care”, the statement was rather assigned to the category “planning of care”. Also, assignment of statements to several categories or multiple coding of one statement was considered possible. During comprehensive quality control, AL reviewed all codings initially assigned by JZ and vice versa. Afterwards, AL and JZ discussed the codings and coding scheme until consensus was found. Qualitative analysis was performed using MAXQDA 12 (Verbi GmbH).

For demographic data and results of the rating, we calculated descriptive statistics using SPSS (IBM SPSS Statistics, Version 23).

## Results

### Description of data sets and sample characteristics

In total, *n* = 18 participants took part in two expert workshops. One participant refused to provide his or her socio-demographic data (except of “current profession”, which was known from all participants). All participants were German native speaker and had a university degree. 88.9% of the participants defined themselves as female, 27.8% were between 50 and 59 years old, 55.5% were non-denominational and 66.7% work in a city with more than 100.000 residents. Eight participants work as gynecologists in practices, nine work as counselors in social support service. One participant is a lawyer working in the executive board of a national provider of social support service centers and has no active part in counseling. Most participants work in different institutions and in different cities and states in Germany, However, most participants work in north or central Germany, were there is (in general) better access to abortion care due to a higher density of abortion care providers [8]. 38.9% of all participants stated to have good and 11.1% stated to have very good knowledge about person-centeredness. Participants stated that they have good (27.8%) or very good knowledge (61.1%) about the topic of unintended pregnancy. A detailed overview on demographic data can be found in Table 1.

**Table 1:** Demographic data of experts of expert workshop

	***n***	***%***
**Total number of participants**	18	100
**Age**^a^		
18–29 years	1	5.5
30–39 years	3	16.7
40–49 years	3	16.7
50–59 years	5	27.8
60–69 years	4	22.2
70–79 years	1	5.5
Missing	1	5.5
**Gender**		
Female	16	88.9
Male	1	5.5
Missing	1	5.5
**Mother tongue**		
German	17	94.4
Missing	1	5.5
**Confession**		
Roman-catholic	2	11.1
Evangelical-lutheran	5	27.8
Non-denominational	10	55.5
Missing	1	5.5
**Current Profession**^b^		
Gyneacologist	8	44.4
Counselor / social educator	7	38.9
Counselor / psychologist	2	11.1
Lawyer working in an executive board of a counseling provider	1	5.5
**Size of workplace**		
< 5.000 residents	1	5.5
< 100.000 residents	2	11.1
> 100.000 residents	12	66.7
Missing	3	16.7
**Knowledge about person centeredness**		
Not at all	2	11.1
Hardly	1	5.5
Some	4	22.2
Good	7	38.9
Very good	2	11.1
Missing	2	11.1
**Knowledge about unintended pregnancy**		
Not at all	0	0.0
Hardly	0	0.0
Some	0	0.0
Good	5	27.8
Very good	11	61.1
Missing	2	11.1
**Work experience**		
Mean (SD)	22.62 (12.81)	
Range in years	2 – 40	
Missing	1	

One participant refused to provide his or her socio-demographic data and was defined as missing in Table 1 (except of “current profession”, which was known from all participants)

*SD* standard deviation

^a^Age was assessed in categories of about 10 years each within the working age range to allow description of the sample without compromising anonymity;

^b^more than one answer possible

The four subgroups of the expert workshops comprised each 4 or 5 participants. Duration of the discussions within subgroups lasted from 77 to 95 min (mean 86 min). For time reasons, not every PC dimension was discussed as initially planned and five to eight dimensions were discussed per subgroup. The dimensions “person-oriented characteristics of healthcare providers” and “team work” were not specifically discussed in any of the subgroups. For an overview on the discussed dimensions per subgroup, please see Table 1 in Additional file 3. Nevertheless, all 16 deductively generated categories were found in the data, even though there were differences in the amount of time available for discussing the single dimensions and two dimensions, which were not discussed at all.

### Results of qualitative expert workshops on relevance and actual implementation of dimensions of person-centeredness in abortion care

**Fig. 1:**
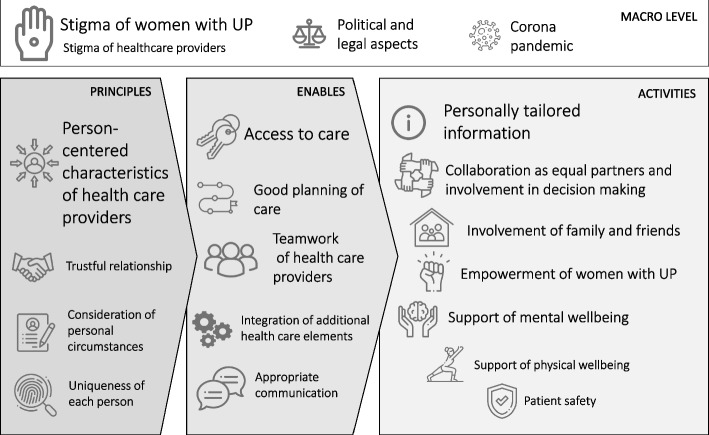
Overview on all major categories described for the three subgroups “principles”, “enables”, and “activities” as well as major inductive categories identified on a macro level. Size of letters reflect discussed relevance of the categories

In the following section, we summarized deductive and inductive categories as results of the qualitative content analysis. Title of main categories are written in bold, titles of subcategories (which are all inductive categories) are written in italic letters. In the following, we used the term HCP to comprise gynecologists and counselors as well as other professions involved in care for women with UP. A comprehensive description of all categories including examples of quotes for all categories can be found in Additional file 4. For an overview on all major categories, see Fig. 1.

#### Subgroup “principles” of the integrative model of PC

*“I think for the vast majority of women, access to information and being generally humane and respectful would probably be enough.” Category “Person-centered characteristics of healthcare providers”, W1G1TN08, Counselor*

*“But in some cases the women are, well, - are given information that is not correct at all. So that would have to be the first thing to change. That can’t be that the professional in charge has no idea about that [abortions].” Category “Competence and informedness of gynecologists”, W2G2TN14, Gynecologist*

Participants of workshops defined a respectful and appreciative behavior of HCPs in interaction with women with UP as crucial **person-centered characteristic of HCPs**. Those characteristics involve to provide space for emotions and a friendly atmosphere, to take the women with UP seriously, to offer neutral, non-prejudiced treatment and counseling based on the individual needs of the women. However, it was reported that gynecologists, who do not perform abortions themselves, often do not know enough about abortion methods (see category “*lack of training of gynecologists*”) and do not perceive offering abortions as a normal and important part of the work of gynecologists (see category “*competence and informedness of gynecologists*”). At most medical schools, abortion methods are not part of the curriculum and gynecologists later have to teach themselves on abortion methods. Participants of workshops therefore highly requested an integration of practice of abortion methods in the medical curriculum. Participants underlined the importance of a respectful attitude of the HCP towards women with UP to enable a **trustful relationship**. This was reported to involve that every women with UP is seen as a **unique person** with individual needs and wishes, especially regarding their pregnancy, their need for more or less information in counseling or their preference for an abortion method. Additionally, participants stated that it is important to **consider personal circumstances** of the women with UP, especially in counseling. Counselors should ask for their *financial and economic situation*, their *partnership*, their relationship to or *involvement of family and friends* and their *cultural and religious background*. Those questions were perceived as important because for example, the financial situation could influence the choice of the abortion method (costs differ between methods). A lack of support by partner or family could influence the decision and could be a barrier for utilization of abortion care.

#### Subgroup “enables” of the integrative model of PC

*“But it [abortion care] is not given throughout Germany. Germany-wide, I see the situation as very, very problematic. It is, yes, difficult in large areas of Germany [...], [facilities for abortion care are] hundreds of kilometers away. And [this is] very difficult for the women to organize.” Category “Access to care”, W2G1TN12, Counselor*

*Persons with UP sometimes wait up to five weeks, because they are held up by their own gynecologist, who doesn’t have the information about how the consultation works and so on. [...] sometimes even the gynecologists don’t know how a medical abortion works. “Category “Access to care in time”, W2G2TN18, Gynecologist*

According to our participants, **appropriate communication of HCPs** furthermore includes creation of a friendly atmosphere, an open-minded and objective communication addressing individual differences as well as fears and worries of the women with UP. Additionally, **integration of additional healthcare elements** (like medical follow-up appointments or additional counseling offers) were observed to be helpful for women with UP. However, participants perceived a lack of specific psychological counseling for women with UP or women who underwent an abortion. Our participants also criticized that **communication and teamwork with HCPs** who do not provide or decline abortion care is often a challenge due to prejudices of some gynecologists. For follow-up appointments, participants reported to rather refer women to gynecologists having positive attitudes towards abortion. That some hospitals refuse to conduct abortions or to treat women with complications after an abortion was described as a major barrier in abortion care in Germany. In our expert workshops, the most intensively discussed dimension of the integrative model of PC was **access to psychosocial and medical abortion care**. Our participants discussed that a fast and low-threshold access to abortions is crucial to person-centered abortion care. Women should receive as much or as less counseling as they need for their decision. However, in many regions in Germany, especially in rural areas and catholic regions in the south of Germany, access to high quality abortion care was described as limited due to a lack of abortion providers. In those areas, women with UP have to travel several hundred kilometers for pregnancy conflict counseling or the abortion. Participants of our workshops claimed that women with UP often have to pay for their abortion and costs differ between abortion providers and abortion methods, which might reflect *financial interests of some gynecologists*. Higher flat rates for abortions using full anesthesia compared to local anesthesia were discussed as one reason for a decrease of practices offering abortions under local anesthesia. Participants reported about *delayed access to abortion due to waiting for health insurers to cover the costs* for the procedure. Thus, participants of our workshops requested that abortions should be accessible within public healthcare without additional costs and that abortions should be regularly covered by the health insurances. Access to abortion care was reported to also include *access to information on different abortion methods and the opportunity to choose between different methods* as well as *access to information about practices offering abortions*. Participants reported that due to restrictions of §219a StGB (prohibiting HCPs to offer information on abortion methods and conditions, paragraph deleted in June 2022 by the German government) those kind of information are often not easy accessible or provided by HCPs. During the discussion, participants highlighted advantages of and access to tele-medical counseling and abortions, especially during restrictions of the Covid-19 pandemic. In Germany, *pregnancy conflict counseling via video or telephone* was first made possible during Covid-19 pandemic. It was reported to simplify the integration of the mandatory counseling appointment in the everyday life of women with UP and to avoid long travel distances. *Medical abortion, applied in home-use,* would ensure a fast and easy access to abortions and would reduce barriers, complications and risks. Especially during the Covid-19 pandemic, tele-medical counseling and medical abortion in home-use were reported to be offered and used more often. However, according to our participants, many HCPs do have a lack of knowledge and reservations about tele-medical counseling and medical abortions, which might be explained by a lack of trust on women using those methods conscientiously and the unclear legal situation. However, participants of our workshops demanded that abortion care should be *accessible in time* and *close to home*. They observed that a severe lack of knowledge of some gynecologists as well as women with UP themselves often lead to a delayed access to abortion care. For example, there might be the false assumption that determine the pregnancy by a gynecologist is necessary before attending pregnancy conflict counseling. Furthermore, *language barriers* might limit access to information about abortion (methods) according to our participants. This problem seem to be increased due to a *lack of suitable interpreters*, which for example have to be engaged and payed by social support services. They also mentioned that *access to medications* as well as *access to contraception* should be regularly integrated in abortion care. Regarding the dimension **good planning of care** of the integrative model of PC, participants highlighted the importance of continuity of care within social support services and practices as well as communication and cooperation between social support services and practices. This would include *sufficient time for counseling and medical consultations*. Regardless of their final decision, women with UP should be offered *follow-up appointments* for counseling and medical care, where there should be space to also discuss other relevant topics like contraception.

#### Subgroup “activities” of the integrative model of PC

*“That often results - in shame or this - oh God, oh God, what happens to me now - that often results from a high lack of knowledge. So predominantly also with young or much younger women, girls, who have this lack of knowledge about what actually happens during an abortion or she has read very terrible things on the Internet. It's like a black hole or a mountain in front of her.” Category “Information on the process of an abortion / legal regulation”, W1G2TN11, Counselor*

*“[Counselors / physicians should convey in conversation,] That this is normal, to have feelings of conflict that actually seem to be in conflict, that at the same time you’re relieved and yet you're terribly sad and just ashamed and so on and so forth.” Category “Empowerment of the person”, W2G1TN13, Gynecologist*

Participants of expert workshops identified receiving **personally tailored information** about abortion and abortion methods as one of the most relevant dimensions of the integrative model of person-centeredness for abortion care [16]. Participants discussed that *manipulation and* (conscious or unconscious) *misinformation* of women by HCPs is not rare in German abortion care and leads to feelings of stigmatization, shame and guilt in women seeking an abortion. Personally tailored information should include *information on the process of an abortion and underlying legal regulations* (e.g., handing over addresses of gynecological practices offering abortions), *information on (medical) consequences of an abortion* (e.g., addressing fears of not being able to become pregnant anymore) as well as *information on contraception and sex education* (e.g., informing about suitable contraceptive methods and strengthening of body awareness). Furthermore, participants discussed that *receiving sufficient and correct information* is essential but often not given. They explained that single gynecologists (intentional and non-intentionally) provide wrong, misleading or manipulative information on the abortion procedure (e.g., suggesting that observing heart tones of the fetus is a necessary requirement for pregnancy conflict counseling).

Receiving sufficient and neutral information about their situation and options were discussed as crucial basis for the **active involvement of women with UP in decision-making**. This includes the availability of different options (e.g., abortion methods) and the possibility to choose between those options, which is often not given in German abortion care. Participants supported the **involvement of family and friends** of women with UP in the decision-making process or a *non-involvement of family and friends* according to the preference of the women. Participants concluded that **empowerment of women with UP** in their ability and responsibility to self-manage their situation, *to make a self-determined decision* that feels right for them and to consider their own life as valuable should be an aim of abortion care. According to our participants, gynecologists should provide **support of mental wellbeing**, for example by providing time, space, being empathic and especially *addressing possible ambivalent feelings* (e.g., to be relieved and sad at the same time after an abortion). In fact, participants observed that many women experience **shame and guilt **when seeking an abortion. This might be followed by self-punishment, e.g. by not taking pain medication and withstand pain due to the abortion procedure. The dimension **support of physical wellbeing** was not in the focus of the discussions within the expert workshops. Nevertheless, they discussed the importance of *pain management*, since this might vary largely between individuals. Participants pointed out that gynecologists have to be aware that some women after an abortion might withstand pain without taking medication to punish themselves. Participants discussed that **patient safety** can be increased by conducting medical abortions, where an intervention in the women’s body is not needed, by handing over the gynecologist’s private phone number to be reachable in case of complications after an abortion and by *privacy protection* (e.g. enabling confidentiality).

#### Inductive categories on the macro level

*“When I talk to young women now in counseling, they say much, much more often than seems plausible to me - I can’t talk to anybody about that there. So I think that not wanting to talk about it or being able to or thinking you couldn’t, that that’s actually rather increased compared to 40 years ago.” Category “Stigmatization of women with UP”, W2G1TN08, Counselor*

In our data, we identified several inductive categories, which are not yet represented in the integrated model of PC and cannot be placed as sub-categories of the existing dimensions [16]. They are more likely additional dimensions on a macro level, which affect several dimensions of the three dimension groups of the integrative model of PC (enablers, principals, and activities). One of the most intensively discussed category on the macro level, which is quite specific for the context of abortion care, is **Sstigmatization of women with UP**. Stigmatization was largely observed in abortion care (i.e. by gynecologists when first determining the pregnancy), in the women’s social environment and in society (i.e. by psychologizing abortions). To foster *de-stigmatization and de-tabooing* of abortions, participants of workshops requested perceiving abortions as a normal and important part of the gynecological practice and offering objective treatment of women with UP in this emergency situation. Participants pointed out that an *unintended pregnancy can happen to every women*, regardless of their education, socio-economic status, or age. On the other hand, also **abortion providers reported stigmatization** and rejection by other physicians. Consequences of **political and legal aspects** on person-centered abortion care were discussed in the context of many of the previously described dimensions. Due to restrictions of §219a StGB [paragraph deleted in June 2022 by the German government], women with UP were reported to have difficulties to find information about practices offering abortions and abortion methods [37]. Additionally, the mandatory pregnancy conflict counseling was perceived as coercion in case of an already clear decision for an abortion. Furthermore, due to the German law defining abortions as a criminal offense, which is not punished under certain prerequisites, gynecologists offering abortions reported to feel like working in a “gray area”. The German law regulates the *right of gynecologists to refuse abortions* except when the women is in danger of death or serious health damage (§ 12 SchKG) [11]. That allows gynecologists even to refuse treatment of women with complications after an abortion. Participants of our workshops discussed that these regulations make it even more difficult to find gynecologists or hospitals performing abortions or after-care, especially in rural or southern catholic regions. Furthermore, participants discussed **influences of the Covid-19 pandemic** on person-centered abortion care. As negative consequences of the pandemic they mentioned higher barriers to access social support services or practices providing abortions and delays in care provision due to closed offices of health insurances which made it difficult to apply for cost coverage. Positive consequences included the increased offer and use of tele-medical pregnancy conflict counseling or medical abortions.

### Results of quantitative rating of relevance and actual implementation of dimensions of person-centeredness in abortion care

Result of the quantitative rating of relevance and actual implementation of the 16 dimensions of the integrative model of PC by participants of expert workshops are displayed in Table 2. Those include values for range, median, mean, standard deviation (SD) and distribution of ratings on the 10-point rating scale. Each dimensions were rated by at least 13 participants of the survey.

**Table 2:** Results from quantitative ratings of relevance and actual implementation of the 16 dimensions of the integrative model of patient-centered care for women with unintended pregnancy by participants of expert workshops

**Dimension**	**Relevance**								**Implementation**							
	**n**	**Range**	**Median**	**Mean**	**SD**	**Distribution of ratings (%)**^a^			**n**	**Range**	**Median**	**Mean**	**SD**	**Distribution of ratings (%)**^a^		
						**1–4**	**5–7**	**8–10**						**1–4**	**5–7**	**8–10**
**Principles**																
Person-oriented characteristics of healthcare providers	15	8–10	10	9.67	0.72	-	-	100.0	14	3–9	7	6.27	2.02	20.0	46.7	33.3
Trustful relationship	14	8–10	9	9.21	0.70	-	-	100.0	14	4–8	6	6.14	1.41	14.3	64.2	21.4
Uniqueness of each person	15	7–10	10	9.33	1.05	-	13.3	86.7	15	4–9	6	6.47	1.69	13.3	53.3	33.3
Consideration of personal circumstances	15	7–10	10	9.13	1.06	-	6.7	93.3	15	2–8	6	5.53	1.64	20.1	73.4	6.7
**Enablers**																
Appropriate communication	15	7–10	10	9.6	0.83	-	6.7	93.3	15	3–8	6	5.67	1.84	33.3	40.0	26.7
Integration of additional healthcare elements	14	1–10	7	6.29	2.95	21.4	42.8	35.7	14	1–9	4.5	4.36	2.06	50.0	42.9	7.1
Teamwork of healthcare providers	15	5–10	10	8.8	1.57	-	20.0	80.0	14	2–9	6.5	6.21	1.97	14.2	50.0	35.7
Access to care	15	8–10	10	9.8	0.56	-	-	100.0	15	2–6	4	4.0	1.56	60.0	40.0	-
**Good planning of Care**	14	6–10	9.5	8.86	1.41	-	21.4	78.6	14	1–9	6.5	6.21	2.33	21.3	42.8	35.7
**Activities**																
Personally tailored information	14	6–10	10	9.29	1.20	-	7.1	92.9	14	2–9	5.5	5.93	2.17	21.3	50.0	28.6
Collaboration as equal partners and involvement in decision-making	15	6–10	10	9.13	1.19	-	6.7	93.3	14	2–9	6	5.93	2.20	28.5	42.9	28.6
Involvement of family and friends	15	4–10	9	8.47	1.73	6.7	20.0	73.3	15	3–10	6	6.47	1.89	13.4	53.3	33.4
Empowerment of the person	15	6–10	10	9.2	1.32	-	13.4	86.6	15	1–9	6	5.53	2.48	33.4	46.7	20.0
Support of mental wellbeing	14	8–10	10	9.36	0.84	-	-	100.0	14	3–9	6.5	6.5	1.61	7.1	71.5	21.4
Support of physical wellbeing	14	6–10	9	8.64	1.22	-	14.2	85.8	13	3–9	6	6.08	1.98	23.1	46.2	30.8
Patient safety	15	8–10	10	9.87	0.52	-	-	100.0	15	4–10	9	7.87	2.00	6.7	26.7	66.6

^a^Distributions of ratings (%) of the consolidated ratings of 1–4, 5–7 and 8–10 of the 10-point rating scale. Rating scale for relevance: from “not relevant” (1) to “extremely relevant” (10), rating scale for implementation: from “not implemented at all” (1) to “largely implemented” (10)

All dimensions were rated as highly relevant with mean values between 6.29 (2.95) (“Integration of additional healthcare elements”) and 9.87 (0.52) (“Patient safety”).

For all dimensions, the maximal value for relevance was rated with 10. However, the dimensions “Involvement of family and friends” (range 4–10, mean: 8.47 (1.73)) and “Integration of additional healthcare elements” (range: 1–10, mean: 6.29 (2.95)) were rated as less relevant from some participants.

For rating of actual implementation of the 16 dimensions, data were much more diverse.

Means varied between 4.0 (1.56) for the dimension”Access to care” and 7.87 (2.00) for the dimension “Patient safety”. Minimum value for each dimension was between 1 (“Integration of additional healthcare elements”, “Good planning of care”, “Empowerment of the person”) and 4 (“Trustful relationship”, “Uniqueness of each person”, “Patient safety”). Only two dimensions (“Patient safety” and “Involvement of family and friends”) were rated with the maximum value of ten by some participants. The dimension “Access to care” was rated with the lowest maximum of 6.

## Discussion

The qualitative study at hand aims to evaluate the relevance and actual implementation of dimensions of PC in psycho-social and medical abortion care from the view of abortion care providers. In qualitative workshops, gynecologists performing abortions and counselors offering pregnancy conflict counseling discussed dimensions of the integrative model of PC [16]. All 16 dimensions of the model were found in the data even though time for discussing dimensions differed between workshop subgroups and two dimensions were not explicitly discussed at all. 31 new categories were found and integrated as subcategories for the 16 dimensions of the integrative model of PC. Furthermore, we found four main inductive categories with three inductive subcategories, that could not be integrated in the original PC model and were therefore organized on a new macro level.

### Most relevant PC dimensions in abortion care and applicability of the integrative model of PC to abortion care

The most intensively discussed dimensions of the integrative model of PC were (in the order in which they are mentioned in the results section): 1) “Person-centered characteristics of HCPs” including their competence and informedness 2) “Access to care” including e.g. access to information of abortion methods, practices offering abortions, tele-medical offers, medical abortions and care without a financial burden or time delay and close to home, 3) “Personally tailored information” including sufficient and correct information on the abortion process avoiding manipulation and misinformation, and 4) “Empowerment of women with UP” to ensure encouragement and a self-determined decision. Further intensively discussed topics, which are not part of the model, were 1) positive and negative influences of the “COVID-19 pandemic”, 2) “Political and legal aspects of abortions” and 3) “Stigmatization of women with UP”. Those did not fit the model and have been assigned to the macro level.

During the process of content analysis, it became apparent that the dimensions of the integrative model of PC in the context of abortion care are strongly interdependent and cannot clearly be separated. For example the observed widespread lack of knowledge of gynecologists on abortion procedures was described to favor negative attitudes towards abortions, less person-centered characteristics of HCPs, less appropriate communication, less teamwork of HCPs, limited access to care and personally tailored information, and more stigmatization of women with UP. Similarly, restrictive healthcare policies and legal restrictions in Germany were discussed as highly associated with a limited access to abortion care and personally tailored information, less person-centered characteristics of HCPs and higher stigmatization of women with UP and abortion care providers as well as increased feelings of shame and guilt of women with UP. However, those interconnections between the dimensions have already been reported by at least one other study in the context of cancer care [38]. The meaning of those interconnections for different healthcare settings should be further evaluated.

In the quantitative rating, all 16 dimensions of the model were rated as highly relevant but most of the dimensions were rated as moderately implemented in German abortion care. This is in line with findings of Zeh et al. (2019) who found limited implementation of the 16 dimensions in healthcare from the view of patients with chronic diseases [17].

Our results indicate that the integrative model of PC can also be applied in the context of abortion care. However, several inductive categories like “stigmatizing of women with UP”, “manipulation and misinformation”, “shame and guilt” and “legal and political restrictions” were discussed as highly relevant in the context of abortion care since those aspects hinder the improvement of the implementation of PC into abortion care in Germany. However, in other fields of healthcare this dimensions might be less relevant. The integration of those inductive categories and the adaptation of the integrative model of PC to the context of abortion care is therefore highly needed to provide a complete picture of PC in German abortion care. 

The macro-level category “corona” has been discussed as both a barrier and a facilitator, for example, through easier access to telemedicine. However, this category is limited to the specific period of the COVID-19 pandemic. A deeper understanding of stigmatization, especially of women with UP, and the relation to “political and legal aspects” is necessary, and further research is indicated when adapting the model and discussing barriers in current abortion care. Moreover, these categories were not part of the quantitative rating in this study. As this study was labeled as a pre-phase, this is planned for the 2nd and 3rd phases of the CarePreg project.

### Barriers for high-quality abortion care in Germany

In our expert workshops, participating gynecologists and counselors draw a devastating picture of the current abortion care situation for women with UP in Germany. Deficits and barriers were described for all dimensions of the integrative model of PC [16]. Access to abortion care including information on abortion methods, processes and legal regulations as well as provision of social support service and medical abortion services close to home, was described as highly limited with high regional differences. Access was primarily described as limited in rural areas compared to bigger cities as well as in catholic regions in south Germany (e.g. Bavaria) compared to more liberal regions in north and east Germany. Furthermore, stigmatizing of women seeking an abortion and of gynecologists providing abortions is still present in German healthcare even though the political agenda, legal restrictions and the societies view on women’s rights and women empowerment became less conservative within the past decade. In fact, restrictive attitudes towards abortions increased within the last 30 years and were more likely in regions with more barriers to access abortions [4]. Thus, stigmatization is still very relevant since it impacts women’s abortion experiences and is a barrier for person-centered care [19]. Our results are in line with other results regarding abortion care barriers [23, 39]. In their study, Summit & Lague (2020) found that many physicians were motivated to provide abortion care but only a minority did so [39]. Barriers to accessible abortion care were legal, political, institutional and funding restrictions, religious affiliation, lack of colleagues' support and stigma of abortion providers [39, 40]. In most highly developed Western-European countries, women with UP and women seeking an abortion still have to cope with a lack of scientifically sound information on abortion methods and procedures, a challenging search for adoption care providers and misleading information of anti-abortion initiatives [19]. In Germany, the high need for an improvement of accessibility of abortion care is also indicated by the decreasing number of practices and hospitals providing abortions and the proportion of women who have to travel to another federal state to have an abortion [41].

The limited knowledge on abortion care among gynecologists was discussed as barrier for high-quality abortion care in our workshops as well as in several international studies [42–44]. In a study by Anderson & Cowan (2021), 53% of the participating physicians, who care for women needing an abortion, do not know how and whom to make referrals for an abortion [45]. Physicians in earlier stages of their career who were not trained in abortion care during medical education, were less familiar with those referral procedures [45]. Congruently, training on abortion care (methods) was discussed as important factor to increase abortion care access in our workshops. In Germany, in the last years, some efforts were made to integrate abortion care in medical education and the first medical guideline on abortions in the first trimester was recently published [46, 47]. Still, participants of our workshops concluded that education on abortion (methods) for medical students and gynecologists has to be further promoted to reduce prejudices and condemnation of women with UP. They call for an integration of abortion care in all medical curricula and to foster abortion care as normal and essential part of the gynecological profession.

Furthermore, participants of our workshops discussed benefits of the integration of telemedical services in abortion care to improve access and PC in abortion care. In line with previous studies, participants highlighted the advantages of pregnancy conflict counseling via telephone or video calls [48–50]. In line with our results, those studies described benefits of telemedical pregnancy conflict counseling including reduced stigma, logistical burden, costs, travel distances as well as saving of time and maintaining of privacy [51, 52]. Additionally, our participants discussed the positive impact of medical abortions on PC in abortion care. Medical abortions have a low risk and are easy accessible and thus promote empowerment of the women with UP by encouraging to self-determined abortions according to their own needs. In international studies, women and HCPs described medical abortion as a less invasive, safe and easy affordable method [53]. However, HCPs knowledge on medical abortion was found to be rather low [54]. In a recent qualitative study, Razon et al. (2022) described facilitators for US family physicians to integrate medical abortions in their practices: training, administrative and community support, and internal motivation to overcome barriers [40]. Also in in Germany, medical abortions are performed to a lesser degree compared to other countries and with high regional differences [8, 55]. However, the new German medical guideline on abortions describes medical abortions as equivalent method to surgical abortions and endorse the telemedical approach [47].

All of the above discussed aspects of abortion care address aspects of women’s autonomy, which plays a central role in the context of care for women with UP [56]. Autonomy requires both, sufficient social conditions to live with children and access to safe and person-centered abortion care in addition to sexuality education and low-threshold access so safe contraceptives [56]. Based on the results of this study, we can underline the request of Doran & Nancarrow (2015) for increased training for physicians, increased range of abortion service options including telemedical options, clear abortion guidelines and a standardized referral procedure to alternative providers when staff have a moral opposition to abortion [23].

### Strengths and limitations of this study

This study is the first study explanatorily assessing relevance and actual implementation of PC in abortion care in Germany from the view of abortion providers. We recruited HCPs in the context of abortion care with diverse demographic characteristics and professions. We were able to collect a rich data set and could identify all dimensions of the integrative model of PC. The extensive coding system with a total of 54 categories and strong interdependency between categories made data analysis challenging. Quality of data analysis was improved by extensive peer-review within the study team. Data were analysed by two members of the study team whereby one of the two study team members was not involved in data collection and do not know the workshop participants personally. This might ensure an objective view on the data and increases validity of this study. Furthermore, the thorough data analysis via MAXQDA followed an a priori defined data analysis scheme and involved a comprehensive quality control conducted by the two coders, which increases reliability of this study.

However, generalizability within Germany is limited since abortion care differs largely within Germany. We aimed to include participants from different regions in Germany, working in areas with different population intensities since we know that there are much less practices providing abortions in rural areas compared to larger cities. However more than 70% of our participants worked in cities with a population of more than 100.000 and most of our participants were located in the north or central of Germany. None of our participants work in in the south of Germany or in very rural areas. Thus, the sampling procedure could limit a fair representation of the target population. However, most have big catchment areas and regularly offer counseling or treatment for women from more rural areas. Furthermore, our sample was quite homogeneous regarding gender, mother tongue and confession of the participants. This also reduces generalizability and has to be noted when discussing the findings of this study. Additionally, it was not a priori planned to return transcripts to the participants for comments and / or corrections and to invite participants to provide feedback on the findings as recommended by the COREQ checklist, which should be discussed as one limitation of this study.

Due to the specific healthcare system and specific legal and political restrictions in the German Criminal Code, generalizability to abortion care in other countries is not possible. Furthermore, the current German government removed §219a StGB from the German Criminal Code about one year after data collection. This paragraph prohibit HCPs to offer information on abortion methods and conditions. Thus, we expect that the relevance of some discussed barriers within our expert workshops changed since data collection. Nevertheless, we believe that effects of this legal change will not be immanent within such short time periods. Even if provision of information on abortion methods by HCPs is allowed nowadays and a medical guideline on safe abortions was finally published, there are still a lot of severe barriers in abortion care in Germany.

## Conclusion

This is the first study in Germany providing insights into relevance and implementation of PC dimensions in psycho-social as well as medical abortion care. All dimensions of the integrative model of PC were rated as relevant for abortion care in Germany and additional relevant sub-dimensions and dimensions on the macro level could be identified. However, in Germany, high quality PC care for women with UP, particularly for those seeking abortion, is insufficiently implemented through limited access to information, lack of abortion care providers, reservations among HCPs, stigma and manipulation. Women with UP seeking an abortion should be guaranteed full access to person-centered care nationwide. To meet the WHO guidelines on abortions, in Germany care structures should enable abortions as part of the general healthcare. This includes a more easy access to evidence-based information and person-centered abortion care, more education on abortion care for healthcare providers, integration of topics of abortion care in medical schools and promotion of de-stigmatizing actions.

### Supplementary Information

**Supplementary Material 1. **

**Supplementary Material 2. **

**Supplementary Material 3. **

**Supplementary Material 4. **

## Data Availability

Datasets (anonymous transcripts of expert workshops and survey data) of the current study are available from the corresponding author on reasonable request.

## Abbreviations

AI: Anastasia Izotova
AL: Anja Lindig
COREQ: Consolidated criteria for reporting qualitative research checklist
HCP: Healthcare provider
IS: Isabelle Scholl
JZ: Jördis Zill
LR: Lara Reck
PH: Pola Hahlweg
PC: Person-centered / person-centeredness
UKE: University Medical Center Hamburg-Eppendorf
UP: Unintended pregnancy
SchKG: Dt. Schwangerschaftskonfliktgesetz (engl. pregnancy conflict act)
SH: Stefanie Heger
StGB: Dt. Strafgesetzbuch (engl. criminal code)
