# Supplementary material for: Assessment of relevance and actual implementation of person-centeredness in healthcare and social support services for women with unintended pregnancy in Germany (CarePreg): results of expert workshops
Source: BMC Pregnancy Childbirth. 2024 Apr 6;24:247. doi: 10.1186/s12884-024-06453-8 (PMC10998354; doi:10.1186/s12884-024-06453-8)
Supplement: Supplementary file 1 — Supplementary Material 1. [file 12884_2024_6453_MOESM1_ESM.docx]

**Additional file 1: COREQ (COnsolidated criteria for REporting Qualitative research) Checklist**

| Topic | ItemNo | Guide Questions/Description | Reported on page No. |
| --- | --- | --- | --- |
| **Domain 1: Research team and reflexivity** | | | |
| Personal characteristics | | | |
| Interviewer/facilitator | 1 | Which author/s conducted the interview or focus group? | See Data collection (page 6 and 7) and Researcher characteristics (Additional file 2) |
| Credentials | 2 | What were the researcher’s credentials? E.g. PhD, MD | See title page |
| Occupation | 3 | What was their occupation at the time of the study? | See Researcher characteristics (Additional file 2) |
| Gender | 4 | Was the researcher male or female? | See Researcher characteristics (Additional file 2) |
| Experience and training | 5 | What experience or training did the researcher have? | See Researcher characteristics (Additional file 2) |
| Relationship with participants | | | |
| Relationship with participants | 6 | Was a relationship established prior to study commencement? | See Recruitment (page 6) |
| Participant knowledge of the interviewer | 7 | What did the participants know about the researcher? e.g. personal goals, reasons for doing the research | See Recruitment and Data collection (page 6 and 7) |
| Interviewer characteristics | 8 | What characteristics were reported about the interviewer/facilitator? e.g. Bias, assumptions, reasons and interests in the research topic | See Data collection (page 6 and 7) |
| **Domain 2: Study design** | | | |
| Theoretical framework | | | |
| Methodological orientation and Theory | 9 | What methodological orientation was stated to underpin the study? e.g. grounded theory, discourse analysis, ethnography, phenomenology, content analysis | See Data analysis (page 7 and 8) |
| Participant selection | | | |
| Sampling | 10 | How were participants selected? e.g. purposive, convenience, consecutive, snowball | See Recruitment (page 6) |
| Method of approach | 11 | How were participants approached? e.g. face-to-face, telephone, mail, email | See Recruitment (page 6) |
| Sample size | 12 | How many participants were in the study? | See sample characteristics (page 8 and 9) |
| Non-participation | 13 | How many people refused to participate or dropped out? Reasons? | Due to the sampling method and study design, information on reasons for non-participation could not be collected. |
| Setting | | | |
| Setting of data collection | 14 | Where was the data collected? e.g. home, clinic, workplace | See Data collection (page 6 and 7) |
| Presence of non-participants | 15 | Was anyone else present besides the participants and researchers? | See Data collection (page 6 and 7) |
| Description of sample | 16 | What are the important characteristics of the sample? e.g. demographic data, date | See sample characteristics (page 8 and 9) |
| Data collection | | | |
| Interview guide | 17 | Were questions, prompts, guides provided by the authors? Was it pilot tested? | See Data collection (page 6 and 7) |
| Repeat interviews | 18 | Were repeat interviews carried out? If yes, how many? | See Data collection (page 6 and 7) |
| Audio / visual recording | 19 | Did the research use audio or visual recording to collect the data? | See Data collection (page 6 and 7) |
| Field notes | 20 | Were field notes made during and/or after the interview or focus group? | See Data collection (page 6 and 7) |
| Duration | 21 | What was the duration of the interviews or focus group? | See description of data sets (page 8) |
| Data saturation | 22 | Was data saturation discussed? | Due to the study design, data saturation was not discussed. |
| Transcripts returned | 23 | Were transcripts returned to participants for comment and/or correction? | See Data collection (page 6 and 7) |
| **Domain 3: analysis and findings** | | | |
| Data analysis | | | |
| Number of data coders | 24 | How many data coders coded the data? | See Data analysis (page 7 and 8) |
| Description of the coding tree | 25 | Did authors provide a description of the coding tree? | See Results section and Additional file 4 |
| Derivation of themes | 26 | Were themes identified in advance or derived from the data? | See Data analysis (page 7 and 8) and Results section |
| Software | 27 | What software, if applicable, was used to manage the data? | See Data analysis (page 7 and 8) |
| Participant checking | 28 | Did participants provide feedback on the findings? | See Data collection (page 6 and 7) |
| Reporting | | | |
| Quotations presented | 29 | Were participant quotations presented to illustrate the themes/findings? Was each quotation identified? e.g. participant number | See Additional file 4 |
| Data and findings consistent | 30 | Was there consistency between the data presented and the findings? | See Results and Discussion section |
| Clarity of major themes | 31 | Were major themes clearly presented in the findings? | See Results and Discussion section |
| Clarity of minor themes | 32 | Is there a description of diverse cases or discussion of minor themes? | See Results and Discussion section |

Tong A, Sainsbury P, Craig J. Consolidated criteria for reporting qualitative research (COREQ): a 32-item checklist for interviews and focus groups. International Journal for Quality in Health Care. 2007. Volume 19, Number 6: pp. 349 – 357
