# Supplementary material for: Assessment of relevance and actual implementation of person-centeredness in healthcare and social support services for women with unintended pregnancy in Germany (CarePreg): results of expert workshops
Source: BMC Pregnancy Childbirth. 2024 Apr 6;24:247. doi: 10.1186/s12884-024-06453-8 (PMC10998354; doi:10.1186/s12884-024-06453-8)
Supplement: Supplementary file 2 — Supplementary Material 2. [file 12884_2024_6453_MOESM2_ESM.docx]

**Additional file 2: Details on researcher characteristics and collection of quantitative data**

**Researcher characteristics**

AL is a female neurocognitive psychologist (Dipl., M.Sc.), post-doctoral researcher in the field of healthcare research and implementation research with a focus on person-centered care (PC) and shared decision-making, and a trained psycho-oncologist. JZ is a female clinical psychologist (Dipl.-Psych.), post-doctoral researcher in the field of healthcare research with a focus on PC and women’s health, and a licensed psychotherapist. JZ and AL had comprehensive experiences in conducting qualitative interviews, focus groups and qualitative content analysis prior to this study. LR is a female clinical psychologist (M.Sc.) and psychotherapist in training. She was experienced with moderating interviews but a first-time moderator of expert workshops. SH is a female clinical psychologist (M.Sc.) and psychotherapist in training. During data collection and analysis, she worked as student assistant for the CarePreg study and had experiences in taking minutes from meetings and group discussions as well as qualitative content analysis.

**Data collection for quantitative rating of relevance and actual implementation of dimensions of person-centeredness in abortion care**

Participants of the expert workshops rated the 16 dimensions of the integrative model of PC regarding their relevance and actual implementation in German abortion care. Therefore, they received a link to an online survey (SoSci Survey GmbH) via e-mail directly after the workshop. Within this survey, we presented the 16 dimensions of the integrative model of PC to participants and asked for relevance and actual implementation of this specific dimension: “How relevant do you find this dimension for the care of unintentionally pregnant women?” and “How well implemented do you find this dimension for the care of unintentionally pregnant women?”. Those items could be answered on a 10-point-scale from 1 (“not relevant at all/not implemented at all”) to 10 (“fully relevant/fully implemented”). Furthermore, we asked participants about their knowledge of PC (“How familiar are you with the topic of patient-centeredness?”) and about their knowledge of unintended pregnancy (“How familiar are you with the topic of unintended pregnancy?”). Those items could be answered on a Likert scale from 1 (“not at all”) to 5 (“very much”). Additionally, participants were asked if they discussed the dimension during the previous expert workshop, which could be answered on a dichotomous scale.
