# Supplementary material for: Assessment of relevance and actual implementation of person-centeredness in healthcare and social support services for women with unintended pregnancy in Germany (CarePreg): results of expert workshops
Source: BMC Pregnancy Childbirth. 2024 Apr 6;24:247. doi: 10.1186/s12884-024-06453-8 (PMC10998354; doi:10.1186/s12884-024-06453-8)
Supplement: Supplementary file 3 — Supplementary Material 3. [file 12884_2024_6453_MOESM3_ESM.docx]

**Additional file 3: Details on expert workshops**

**Details on the two expert workshops**

Table 1: Details on subgroups of the two expert workshops: number of participants, name of moderator, dimensions planned for discussion and finally discussed dimensions.

| **Number of workshop and subgroup** | **Number of participants** | **Moderator** | **dimensionen of the integrative model of PC, planned for discussion in the workshops** |
| --- | --- | --- | --- |
| Workshop 1  Subgroup 1 | n=5 | JZ | Discussed during workshop:  Access to care  Consideration of personal circumstances  Appropriate communication  Support of mental wellbeing  Collaboration as equal partners and involvement in decision-making  Not discussed during workshop:  Person-oriented characteristics of healthcare providers *  Patient safety  Teamwork of healthcare providers * |
| Workshop 1  Subgroup 2 | n=4 | LR | Discussed during workshop:  Personally tailored information  Uniqueness of each person  Empowerment of the person  Support of physical wellbeing  Good planning of care  Involvement of family and friends  Not discussed during workshop:  Trustful relationship  Integration of additional healthcare elements |
| Workshop 2  Subgroup 3 | n=4 | LR | Discussed during workshop:  Access to care  Consideration of personal circumstances  Support of mental wellbeing  Collaboration as equal partners and involvement in decision-making  Patient safety  Not discussed during workshop:  Appropriate communication  Person-oriented characteristics of healthcare providers *  Teamwork of healthcare providers * |
| Workshop 2  Subgroup 4 | n=5 | JZ | Discussed during workshop:  Personally tailored information  Uniqueness of each person  Empowerment of the person  Support of physical wellbeing  Good planning of care  Involvement of family and friends  Trustful relationship  Integration of additional healthcare elements |

* Dimensions were not discussed in one of the workshops
