# Supplementary material for: Assessment of relevance and actual implementation of person-centeredness in healthcare and social support services for women with unintended pregnancy in Germany (CarePreg): results of expert workshops
Source: BMC Pregnancy Childbirth. 2024 Apr 6;24:247. doi: 10.1186/s12884-024-06453-8 (PMC10998354; doi:10.1186/s12884-024-06453-8)
Supplement: Supplementary file 4 — Supplementary Material 4. [file 12884_2024_6453_MOESM4_ESM.docx]

|  | | | | |
| --- | --- | --- | --- | --- |
| **Additional File 4. Description of all codes, qualitative codings and quotes.** | | | | |
| **Notes:** * Description of the first 15 main dimensions (all deductive categories in bold letters) are taken from Zill JM, Scholl I, Härter M, Dirmaier J. Which Dimensions of Patient-Centeredness Matter? - Results of a Web-Based Expert Delphi Survey. PLoS One. 2015;10(11):e0141978. The dimension "Patient safety" was defined by Zeh et al. (Zeh S, Christalle E, Hahlweg P, Härter M, Scholl I. Assessing the relevance and implementation of patient-centredness from the patients’ perspective in Germany: results of a Delphi study. BMJ Open. 2019;9(12):e031741.). | | | | |
| **Code** | **Description*** | **Deductive / Inductive** | **Qualitative analysis** | |
|  |  |  | **Summary of codings** | **Quotes** |
|  |  |  |  |  |
| **Macro level** | | | | |
| **Stigmatization of women with UP** |  | inductive | - According to participants, tabooing abortions increase stigmatization of women with UP   Stigma in abortion care: - Participants discussed that stigmatization already starts at the registration desk in the gynecological practice: women with UP must "out" themselves regarding their unintended pregnancy / desire for abortion. - Participants observed stigmatization by gynecologists, in counseling and in clinics that offer and do not offer abortions.  Stigmatization in the women's environment and in society: - Less exchange on the topic abortion within friends was reported. - Stigma was also reported to be particularly present in the context of certain religious and cultural contexts. - Stigmatization was reported to be increased by psychologization of abortions. | "When I talk to young women now in counseling, they say much, much more often than seems plausible to me - I can't talk to anybody about that there. So I think that not wanting to talk about it or being able to or thinking you couldn't, that that's actually rather increased compared to 40 years ago." *W2G1TN08, Counselor*  "But that there are just multiple ways to live a life for oneself. Whether it is with an abortion or just having the child. So that's what we stand for, that is, for this neutral image of women in society. And to get rid of that, that's really generation after generation [...] And if you don't do that, then that's guilt and shame." *W1G2TN09, Counselor* |
| De-stigmatization and de-tabooing |  | inductive | - Participants perceived an unwanted pregnancy as an emergency for the woman that should be considered objectively. This view would reduce stigmatization. - Participants request gynecologists to consider abortions as an important part of their practice. | "It is just good if a woman can have her child, but the situation is that sometimes she cannot. [...] That is not avoidable. And sometimes women decide to take this way. And it is good if they can built a good way for themselves, can decide on a good way and also have good support. And not being bashed." *W1G2TN09, Counselor* |
| Unintended pregnancy can happen to every women |  | inductive | - According to participants, in counseling, women with UP should be explained that an unwanted pregnancy can happen to anyone. - Physicians often ascribe stereotypical roles and images to women with UP. - In contrast to existing stereotypes, the group of women with UP was found to be very diverse (different age, education, socioeconomic status, etc.) | "It is - anyone can get into such situations. Contraception might not work. Nothing is 100%, [...] Any woman can get into such a situation. Any couple can get into such a situation. It's completely independent of whether she's rich or poor or smart or dumb. So that's just the way it is." *W1G2TN07, Gynecologist* |
| **Stigmatization of abortion providers** |  | inductive | - Participants reported that gynecologists offering abortions are often stigmatized and rejected by other physicians. - Regional differences are reported: in Eastern Germany and in larger cities such as Hamburg and Berlin, abortion was reported to be more socially accepted compared to other regions. | "And the legal aspect, too, of course. So that's also a big uncertainty, all these legal restrictions. That scares a lot of people, because if you don't have practical experience with it, then you won't be able to offer it in practice later on." *W1G2TN06, Gynecologist* |
| **Political and legal aspects** |  | inductive | Participants mentioned several barriers due to the legal situation in Germany:  - Due to restrictions by § 219a and the definition of abortions as criminal offense by law (which will not be punished under certain conditions), it was found difficult to get information about abortions (list of gynecologists offering abortions, information of methods which are offered)  - Performing abortions is often not part of the medical training at universities and is often not taught in hospitals. - Gynecologists providing abortions would often have the impression that they are working in a "gray area". - Counseling is perceived as coercion by the women with UP in case of an already clear decision for an abortion.  Political aspects:  - According to participants, various professional gynecological associations and influential organizations do not support the spreading of information on medical abortion. | "And I have never experienced people so small as [...] in this situation of unwanted pregnancy and when they come to abortion. [...] How am I supposed to empower the women now? [...] And I think society makes women even smaller. And the law makes them small." *W2G2TN15, Gynecologist* |
| Right of gynecologists to refuse abortions |  | inductive | - Participants critized that gynecologists and clinics have the option to refuse performing abortions, some even refuses treatment of women with complications after an abortion and women have to be transferred to another clinic in another town. | "So then it endangers the women, yes. [...] - to move 30 kilometers for an abortion - that's - so it's crazy that that's allowed to happen at all, yes. That the clinics are allowed to refuse. I think that's just impossible." *W2G2TN18, Gynecologist* |
| **Influences of the COVID-19 pandemic** |  | inductive | Negative factors:  - Women with UP were reported to be in an emergency situation and perceived to not be seen by politics during the pandemic. - Contact with social support service centers/practices and search for information were more difficult, resulting in higher barriers to seek help. - Required Corona tests and prohibition of access to facilities by accompanying persons were perceived as additional barriers. - Performing an abortion during quarantine or COVID-19 infection was perceived as difficult. - Closed offices of health insurance companies would have led to more difficult applications for cost coverage.  Positive factors:  - Open telephone hours instead of open office hours in social support service centers were reported to reduced access barriers. > Counseling was easier to integrate into everyday life of women and to meet the pregnant woman's needs (e.g. for confidentiality).  - Because women often had to come alone to the abortion, they had more relief and rest in the recovery room. - Participants welcomed that supply and demand for medication-based abortion options had increased. | "I actually found that COVID-19 was partly an advantage for the women, at least here [...]. And I think that will probably have been the case in most places, [a social support service center] offered pregnancy conflict counseling by telephone and that was for many women, especially where - where in the family, where it was difficult with the planning - whether it was that somehow children were in the household or the partner was not supposed to notice or whatever, the constellation was, that made it easier for the women to simply withdraw somewhere and talk on the phone for a quarter of an hour. And then just to have that done with it." *W1G1TN02, Gynecologist* |
| **Principles** | | | | |
| **Person-centered characteristics of healthcare providers** | A set of attitudes towards the person (e.g. empathy, respect, honesty) and oneself (self-reflectiveness) as well as medical competency | deductive | - Counselors and gynecologists should have person-oriented characteristics like > providing a humane, respectful and appreciative treatment of women with unplanned pregnancy (UP) > providing space for any kind of emotions  > reducing feelings of shame and guilt  > taking women with UP seriously and support them  > responding to individual needs of women with UP - counseling and medical abortion treatment should be unprejudiced - women with UP should be provided time to for decision-making - an atmosphere should be created in which women with UP feel comfortable | "I think for the vast majority of women, access to information and being generally humane and respectful would probably be enough." *W1G1TN08, Counselor*  "I don't think it's my job to question that, but rather I'm there to support this woman and to carry out what she has decided to do. And if I notice she's ambivalent, then [...] give her time to decide again, to come back another day or later." *W1G1TN04, Gynecologist* |
|  |  |  | - According to participants of workshops, there should be a self-understanding among gynecologists that abortions are part of their profession and can be a part of a woman's life - this self-understanding would often not be given. | "[...] it [abortion] is just treated in such a way that it just doesn't happen with a professional distance." *W1G1TN04, Gynecologist* |
| Training of gynecologists |  | inductive | Participants reported a hugh lack of knowledge and lack of training on abortion care within gynecologists: > Instrumental abortions are usually not been taught.  > Due to the lack of training in medical schools and clinics, assistant physicians were found to receive little knowledge on abortions during their medical training. > In some regions, abortion using local anesthesia are offered rarely, because it was offered mainly by older gynecologists, who retire within the next years. - Often, a lack of knowledge was reported in communicating with women of diverse cultural and religious backgrounds and with emotions and fears of women with UP. - Missing knowledge has to be acquired by gynecologists themselves and on their own responsibility.  - But participants also observed some positive changes during the last years (e.g., the topic abortion becomes more present at scientific congresses).  - Participants call for a more intensive integration of the topic of abortion in medical curricula, also to reduce prejudices towards women seeking an abortion. | "And as the colleague just said, sometimes even the gynecologists don't know how a medical abortion works. So those who should actually know - but just because it is not taught. So how are patients supposed to know?" *W2G2TN18, Gynecologist* |
| Competence and informedness of gynecologists |  | inductive | - It was found that many physicians had little contact with women with UP and less knowledge about abortions (e.g., some gynecologists were reported to assume that cardiac activity of the embryo must be evident before an abortion can be done). - Some physicians were also perceived to be inexperienced in communicating with people of other cultures. - One of the reasons for a lack of competence of physicians was reported to be that abortions are not taught in medical school, and junior physicians in hospitals have little experience with the abortion procedure.  - One consequence was perceived to be, that fewer physicians perform abortions and fewer instrumental abortions are performed under local anesthesia (in contrast to abortion under full anesthesia). | "But in some cases the women are, well, - are given information that is not correct at all. So that would have to be the first thing to change. That can't be that the professional in charge has no idea about that [abortions]." *W2G2TN14, Gynecologist* |
| **Trustful relationship** | A partnership with the patient that is characterized by trust and caring | deductive | - The goal was reported to be a respectful relationship between the counselor/physician and the person with UP.  - There should be a permanent contact person for the person with UP during the abortion procedure in the practice, this would enable building of trust  - It would also be helpful to create an atmosphere in which the persons with UP felt comfortable (design of the premises, manner of greeting). | "[...] that a woman or a couple can open up in terms of the subject matter, that's simply the most important thing at the beginning and that's where it starts with us [the physicians] somehow also with the - with the facilities or with the manner of the welcome or so - it already starts, so that you simply somehow create an atmosphere that - that the women can feel comfortable [...] - that there doesn't have to be any fear." *W2G2TN17, Counselor* |
| **Uniqueness of each person** | Recognition of each patient’s uniqueness (individual needs, preferences, values, feelings, beliefs, concerns and ideas, and expectations) | deductive | - Participants stated that women with UP should be seen in their entirety not only as a person with wishing to have an abortion. - Each woman should be respected with her individual demands for counseling, her attitude towards her pregnancy, her wish for knowledge, and her own needs, values and preferences. | "What I really wanted to say was that [during abortion care] you have to adjust to a lot of situations yourself. [...] You can't do everything according to a pattern. You just have to feel out a little bit what the patient is looking for." *W1G1TN02, Gynecologist* |
| **Consideration of personal circumstances** | Recognition of the patient as a whole person in his or her biological, psychological, and social context | deductive | - Individual circumstances and situations in life (e.g., financial situation, access to knowledge about contraception, partnership) should be addressed in counseling. - Participants reported that addressing those topics could have an impact on the choice of abortion method (e.g., preference for medical abortion at home at the weekend to not miss work). | "[...] - the psychological dimension of the two methods [medical and surgical abortion] is relatively different. And whether one suits me or the other - I think that has to be discussed with the woman, just by also describing the processes [...]" *W2G1TN13, Gynecologist* |
| Consideration of the financial and economic situation |  | inductive | - According to our participants, a woman's financial and economic situation should be addressed in counseling, as it affects  > the choice of abortion method, as costs vary for different methods > access to contraception  > the decision to terminate the pregnancy or to carry the pregnancy to term | "And of course that's clear, there's the one who can't afford it and she comes in because she doesn't have an insurance at all and she takes the cheapest option." *W1G1TN02, Gynecologist* |
| Consideration of partnership / relationship |  | inductive | - Partnership / relationship was perceived to influence:  > the wish for abortion (e.g., because the partner do not want to have a child, partner puts the pregnant women under pressure) > (emotional) support from partner during pregnancy and/or abortion - But according to participating counselors, problems in the partnership can hardly be addressed properly or solved during pregnancy conflict counseling. | "Then, of course, with women, many, many other things come into it. Like cultural things, power relations between husband and wife, and yes. Or that the mother-in-law decides how many children the woman has. That is then actually also an unwanted pregnancy." *W1G1TN09, Counselor* |
| Involvement of family and friends |  | inductive | - According to participants, counselors should address support by family and friends in counseling (e.g., women might experience exclusion and pressure by their family for cultural or religious reasons). - Regional differences regarding the involvement of friends and family was reported, but the general experience of the participants is that many women with UP talk bout their situation with others. | "There's a lot of backgrounds and a lot of ways where women are also marginalized or pressured by families or by the environment or the idea, actually, in any case, it's the better decision if you carry the pregnancy to term, if you can somehow manage that." *W2G1TN11, Counselor* |
| Consideration of cultural or religious background |  | inductive | - Participants observed that women from Islamic families were often afraid that the family would find out about the unintended pregnancy or abortion. - Knowing the cultural background of the woman with UP as well as addressing sexual violence was reported to be important for counseling and might influence the decision for an abortion method. | "And I found that interesting again, that she [another gynecologist] also looks so little at what is behind it [the wish for an abortion] and perhaps asks herself whether this woman herself finds it great or whether she perhaps does it for certain reasons or out of a certain social background. [...] So I also had the feeling that this [the abortion] is judged [by the gynecologist] a bit quickly." *W1G2TN06, Gynecologist* |
| **Enablers** | | | | |
| **Appropriate communication** | A set of verbal and nonverbal communication skills | deductive | According to our participants this dimension involves: > creating a friendly atmosphere for the conversation in counseling and the gynecological practice > offering unbiased and objective counseling > considering individual differences (e.g., preference for the degree of (emotional) distance in counselling) > appropriate reaction and response to fears and concerns | "And then also really, I think, if these fears are taken away, that the women would have to perform something [...], then the way is open [for the women with UP] to address fears and worries or just to say, no, I don't want to talk about it there." *W1G1TN05, Counselor* |
| **Integration of additional healthcare elements** | Recognition and integration of non-medical aspects of care (e.g. patient support services) into health care services | deductive | - According to participants, this involves referring to other social support services for a more specialized and comprehensive psychological counseling or treatment. - Additional healthcare offers were found to be less present in abortion counseling and care because they are often not available. - Participants indicated that additional healthcare offers have to be available to be recommended by counselors and gynecologists. | "Well, that there are these offers also, because clearly it must be our professional impression - they [person with UP] need more than a sympathetic, basic psychosomatic primary care now. And then [the question is] is it available - this care, yes?" *W1G1TN05, Counselor* |
| **Teamwork of healthcare providers** | Recognition of the importance of effective teams characterized by a set of qualities (e.g. respect, trust, shared responsibilities, values, and visions) and facilitation of the development of such teams | deductive | Cooperation between gynecologists: - Participating gynecologists reported to offer follow-up or after-care after an abortion in their own proactive or to recommend gynecologists who are known to have a positive attitude towards abortions (otherwise women with UP would have limited access to gynecologists for follow-up or after-care). - Due to missing and wrong knowledge among gynecologists it was reported that networking and an offensive appearance of gynecologists offering abortions is important. - Exchange between colleagues (gynecologists and counselors) was perceived to be important to ensure continuity of care. | "So you have to go on the offensive and you have to communicate that to your colleagues - how is it going. Because, unfortunately, medication-based pregnancy termination is not part of gynecologist training at all. And so there are quite, quite a lot of misconceptions among colleagues." *W2G2TN14, Gynecologist* |
|  |  |  | Cooperation between practices and hospitals:  - Cooperation between practices and hospitals was reported to be important but often difficult (e.g., because some clinics would refuse to continue treatment after an abortion in case of complications) | "And then you have to phone your way through it, and somehow it's also constantly changing who's doing abortions now and who's not. And yes, I still find that a bit difficult there, the arrangement." *W1G2TN06, Gynecologist* |
|  |  |  | Cooperation between social support services:  - Especially in counseling, many networks were reported to be available (e.g., family education centers (dt. "Evangelische Familienbildungstätten"), the "early help program" (dt. "Frühe Hilfen"), facilities for mothers with their children (dt. "Mutter-Kind Einrichtungen")) | "Well, my experience is that you also have to maintain the communication between gynecologists, other providers and also us [...] counselors, that information is exchanged about innovations, how the abortion care is handled. That we can also advise the women well." *W1G2TN08, Counselor* |
| **Access to care** | Facilitation of timely access to healthcare that is tailored to the patient (e.g. decentralized services) | deductive | - Participants reported that fast, easy and low-threshold help is necessary - Each person should get as much and as little advice as he/she needs - In many regions of Germany, there a lack of institutions that offer counseling/abortion was observed, persons with UP have to drive many hundred kilometers for counseling / an abortion - Participants reported a lack of access to information on where to go for an abortion - Tabooing of the topic abortions would make access to care more difficult (e.g., persons with UP would often have to mention their unintended pregnancy / wish for an abortion when registering in a gynecologist's practice) - Participants reported the fact that many gynecologists no longer wanted to offer abortions as a main barrier for access to abortion care | "But it [abortion care] is not given throughout Germany. Germany-wide, I see the situation as very, very problematic. It is, yes, difficult in large areas of Germany [...], [facilities for abortion care are] hundreds of kilometers away. And [this is] very difficult for the women to organize." *W2G1TN12, Counselor* |
| Financial interests of some gynecologists |  | inductive | - Abortions in Germany were reported to be associated with higher costs for the women compared to other European countries. - Costs differ dependent on the abortion method applied (e.g., anesthesiologists receive more money for general anesthesia, which is thus provided more often and has an influence on the regional decrease in the offer of local anesthesia). - Participants called for abortions not to be offered for profit. | "Because they [anesthesiologists] simply have a lobby in this area that we don't have. And there is - there it is really simply about money. And politics is also being made. That is - that is crazy. And of course they say - okay, so - you, do it [the abortion] with general anesthesia, otherwise it's terrible and so on. And then you actually earn quite good money with it." *W1G1TN04, Gynecologist* |
| Access to information on different abortion methods / opportunity to choose between methods |  | inductive | - Participants reported that often there is no option for women with UP to decide between several abortion methods because they are not available (e.g., local anesthesia or medical abortions were not or rarely offered in certain regions) - Access to information about different methods was found to be difficult - The financial situation of the women with UP influences the abortion methods, which are affordable for the women | "So that now some of the older physicians are retired and now it is really hard to find [physicians who offer surgical abortions under local anesthesia]. And we still think that this is a very good method. And it would be nice, of course, to have a real choice for women." *W2G1TN11, Counselor* |
| Access to information about practices offering abortions |  | inductive | - According to our participants, women with UP can get information about practices that offer abortions via: > lists provided by counselors > an official list of the German Medical Association (dt. "Bundesärztekammer), but this list is not complete > lists for single cities or regions in Germany (which are partly not complete) - Access to practices offering abortion care was reported to be difficult: > Many physicians do not want to be on an official list to avoid denunciation.  > Some internet website provide partly incorrect, manipulating or incomplete information.  - Access to hospitals for abortions or abortion follow-up care is difficult as some of them refused to provide follow-up care, to treat complications after an abortion, or to perform the abortion. - Participants find it necessary that gynecologists are allowed to provide information about abortion options and methods they offer on their websites. | "And we've also just had the experience during COVID-19, or often anyway, that women come in very late because they've had to work their way through this very non-information jungle first. So it's very far from any form of accessibility, anyway." *W1G1TN01, Counselor* |
| Pregnancy-conflict counseling via video or telephone |  | inductive | - Tele medical services like telephone consultations was told to be increasingly offered through COVID-19 pandemic and improved abortion care in Germany: > According to our participants, women can easier integrate telephone consultations into their daily life compared to visiting a social service center for (e.g., by avoiding long travel distances for pregnancy conflict counseling) | "This [pregancy conflict counseling via telephone] was for many women, especially where - where in the family, where it was difficult with the planning - whether it was that somehow small children were in the household or the partner was not supposed to notice or whatever the constellation was, it made it easier for the women to simply withdraw somewhere and talk on the phone for a quarter of an hour. And then just to have that done with it." *W1G1TN02, Gynecologist* |
| Access to medical abortion in home-use |  | inductive | - According to our participants, access to tele medical services would allow earlier and less complicated abortion with fewer side effects and risks. - Women with UP should receive detailed information about the process, which can be supplemented by apps such as "MedABB" (a German App, which guides the women through the process of medical abortion in home-use and offering support when needed) | "Home use is something that I also offer. That is, most women are also happy to make use of that." *W2G1TN13, Gynecologist* |
|  |  |  | - Participants reported reservations among gynecologists towards medical abortion in home-use, they explained this with a lack of trust in the women - As a consequence, only little information can be found about medical abortions in Germany. | "What is really the dilemma with tele medical abortion is that there are so many fears [of the gynecologists] about offering it, that [...] the women themselves demand it for themselves via social media, because they don't get access o it via the official institutions [...] because there are so many reservations on the part of the professionals, yes." *W1G1TN04, Gynecologist* |
| Access to care in time |  | inductive | - Low-threshold, simple and fast access to abortion care was found to be very important and was defined a goal for abortion care in Germany. - According to participants, no pressure should be put on the woman with UP, even if it can be defined as an emergency situation. - Often, care was reported to be unnecessarily delayed due to a lack of knowledge on both sides, gynecologists and women with UP (e.g., many women with UP have to a long time for an appointment to determine the pregnancy, which is not a necessary requirement for pregnancy conflict counseling). | "Persons with UP sometimes wait up to five weeks, because they are held up by their own gynecologist, who doesn't have the information about how the consultation works and so on. [...] sometimes even the gynecologists don't know how a medical abortion works." *W2G2TN18, Gynecologist* |
| Access to care close to home |  | inductive | - Participants described that there is no regional coverage of abortion care in Germany. - Participants imagined that an increased offer of (tele-)medical care could improve this situation. | "There are areas, rural areas in the federal territory, which are not represented here at all or hardly today. There it is - so there the women have to drive insanely far and are sometimes not even able to drive that far or even be able to drive to another city to do the abortion there." *W1G2TN08, Counselor* |
| Delayed access to abortion due to long waiting for health insurers to cover costs |  | inductive | - The cost coverage procedure of health insurances was reported to delay treatment: > Health insurance companies sometimes offer only late appointments to apply for the cost coverage. > No coverage of costs is possible with women's income slightly above the income limit. > During the COVID-19 pandemic, health insurance offices had been closed, women with UP had difficulties obtaining the necessary application forms in a timely manner. - Thus, participants requested that abortion care should become a normal part of the health care system and costs should be covered by health insurances. - Currently, the prices for abortion in Germany were reported to vary between methods and participants reported that single gynecologists would only accept cash or would not accept cost coverage by health insurances. | "So we want to make sure that financing is not an issue at all, yes. So that women really have this medical offer [...] - like when they want to have their appendix or their ovaries or whatever operated, that they can decide that in the same way. And that really everything [every abortion method] is available, both financially and in terms of expertise." *W1G1TN04, Gynecologist* |
| Limited access due to language barriers |  | inductive | - Language (and digital) barriers would limit access to information, which can lead to losing time in the abortion process | "[...] but there are always some [women] with language problems, with a migration background. Simply women who are not as digitally proficient as perhaps a young student or so. And there is [...] extremely much loss of time." *W2G2TN18, Gynecologist* |
| Lack of suitable interpreters |  | inductive | - Participating counselors found it difficult to recruit suitable interpreters, especially under time pressure. - Interpreter services have to be provided by social support services themselves, often funding for this offer was reported to be lacking. - Some interpreters were found to refuse translating a pregnancy conflict counseling or to be biased. - Often family members of women with language barriers (sometimes the own children of women with UP, younger siblings, etc.) had to translate during pregnancy conflict counseling | "But [...] that's already really hard, then [...] such a 10-year-old boy translates for a pregnancy conflict counseling - so it really just doesn't work at all. [...] And yet it's just reality, what - if there's no one else who can do it, what are you supposed to do?" *W2G2TN17, Counselor* |
| Access to medications |  | inductive | - Medical abortion was found to be a less distributed method in Germany: > It was recently discussed that the drug Cytotec (used for medical abortion) should be taken off the market. > Dissemination of information on medical abortion is partly not supported by gynecological associations and journals. | "And so to not worsen the situation for women [and to prevent Cytotec from being taken off the market], there has been a collaboration of very different organizations. I see that now as a glimmer of hope." *W2G1TN12, Counselor* |
| Access to contraception |  | inductive | - Participants reported that the easy access to contraceptives nowadays is often causing negative attitudes towards women with UP - woman are blamed for not using contraception well enough. - Costs for contraceptives would mostly be covered by the woman. | "And I can tell you, for example, that quite a few of my colleagues have a negative attitude toward abortion because they say there are enough contraceptives available. It's a thing [to become unintendently pregnant] that doesn't have to be today. So a certain kind of blaming the woman." *W2G2TN14, Gynecologist* |
| **Good planning of care** | Facilitation of healthcare that is well coordinated (e.g. regarding follow-up arrangements) and allows continuity (e.g. a well-working transition of care from inpatient to outpatient) | deductive | - Participants stated that in practices, continuity could be achieved by gynecologists being availability and addressable after the abortion procedure. Additionally, they should provide information on after-care - Social support service was reported to cover various counseling topic, even after pregnancy conflict counseling has taken place and the decision was made - women are often not aware about this offer. - Exchange and cooperation between social support service centers and practices can ensure continuity of care. | "Well, it is already part of it that I inform on the one hand, what might happen afterwards. What is normal, what is not normal. And then encourage them [women with UP] to call, or just that I take care that they are in good hands afterwards. [...] that I then say – oh, then go to a colleague who stands also behind the abortion. That is, who accepts it." *W2G2TN14, Gynecologist* |
| Sufficient time for counseling and medical consultations |  | inductive | - Participants argued that it is essential for a good counseling to have enough time to explain the treatment methods and to explore the personal circumstances of the woman with UP. - In Germany, capacities for the entire abortion treatment spectrum were reported to be not available because the number of abortion care providers has decrease and still decreases. | "We are fortunate [in our practice] that we always have half an hour per woman and therefore always [...] offer to talk about contraception and there is often a great need there and some say then, yes, then - so far I have not had the opportunity to talk about it with a physician for so long and there you already notice that so in the normal practice this often falls down. So they don't - they don't discuss all the options, they just quickly prescribe the pill or something." *W2G2TN06, Gynecologist* |
| Possibility for follow-up appointments |  | inductive | - Follow-up appointments in social support service centers and practice might be important to discuss contraception or offer further counseling. - Follow-up care was reported to be often available in practices that also perform abortions. | "The offer of psychological support, that it also includes pregnancy conflict counseling, that one offers that the women can come again and that they can also come, no matter what happens next. [...] No matter whether they decide to stay pregnant now or whether they have an abortion, they always have the opportunity to come to the counseling session." *W1G1TN05, Counselor* |
| **Activities** | | | | |
| **Personally tailored information** | Provision of tailored information while taking into account the patient’s information needs and preferences | deductive | - Participants described access to information and detailed medical education as an essential basis for decision-making. - Participants wanted information about the abortion procedure to be openly available, clearly visible, and easily accessible. | "Often the problem is that the women, even if they are with us, with the physician they trust, who in this context do not receive the information [about abortions], that they do not get it right away. And in many cases only the pregnancy is confirmed [by the gynecologist] and then they are told, yes, see how you can manage this now. Or go to a counseling center." *W2G1TN12, Counselor* |
| Manipulation and misinformation |  | inductive | Participants discussed that misinformation about abortions is spread knowingly or unknowingly:  > Psychologization: wrong assumption that abortions would lead to trauma > Gynecologists make unnecessary appointments for consultations with the women with UP before initiating the abortion (e.g., waiting to hear heart tones of the fetus). > Abortion opponents (in German so called "Lebensschützer") provide fake counseling or websites with manipulative or false information. > Single gynecologists do not inform their patients about the option of cost coverage by the health insurances or only offer abortions for cash. > Single counseling centers would also provide manipulative counseling | "But even there it happens that - yes, that especially older gynecologists also share their own opinion and somehow try to influence the women into one direction. Or the subject of abortion is not even up for debate, or the ultrasound is shown immediately, the picture is shown, the sound is turned on. That there is little sensitivity, so to speak. But that's - yes, in my experience, it's rather a bit occasional." *W2G2TN17, Counselor* |
| Information on the process of an abortion / legal regulation |  | inductive | - Participants reported gynecologists usually only confirm the pregnancy but do not provide information about the whole abortion process, which often leads to a delay in abortion care. - Participants request that addresses of practices offering abortions should always be handed over directly by the gynecologists, who confirms the pregnancy. - Individual information needs should be addressed in counseling and consultations with gynecologists. | "That often results - in shame or this - oh God, oh God, what happens to me now - that often results from a high lack of knowledge. So predominantly also with young or much younger women, girls, who have this lack of knowledge about what actually happens during an abortion or she has read very terrible things on the Internet. It's like a black hole or a mountain in front of her." *W1G2TN11, Counselor* |
| Information on (medical) consequences of an abortion |  | inductive | - Participants observed that some women with UP would raise concerns about medical consequences of an abortion (e.g., fear of being able to become pregnant after an abortion or getting a depression) - Fears should be addressed in counseling and medical consultations, feelings of guilt should be reduced. | "So one question that almost always comes up and is a big concern of the women is - Won’t I be able to have any more children afterwards? [...] - also this information that an abortion is a minor procedure [must be addressed] [...] So that they - that their fear is taken away." *W2G2TN18, Gynecologist* |
| Information on contraception and sex education |  | inductive | - Information about contraceptive methods should be part of the follow-up consultation after an abortion (e.g., to discuss, what women with UP can do to not get into such a situation again) - Aim of follow-up consultations should also be strengthening of body awareness and the perception of the own body. | "[...] and there is often also a great need [for addressing contraception in a consultation] and some then say yes, yes, then - until now I also did not have the opportunity to talk about it with a physician for so long and there you already notice that so in the normal practice that often falls down." *W1G2TN06, Gynecologist* |
| Receiving sufficient and correct information |  | inductive | - Participants reported deficits in provision of information by gynecologists: > Some women with UP reported to being insulted and manipulated by gynecologists (e.g., telling the wrong gestation week) and received incorrect information. > Some women were called in again by the gynecologist until heart tones of the fetus are visible (which is not necessary for an appointment at social support service or the planning of an abortion). > Participants reported confusion about legal restrictions among gynecologists. > Participants reported that some gynecologists suggest that women with UP must have been determined by a gynecologist before they received counseling, which indeed is not necessary.  - Participants mentioned websites on the Internet that provide false information (made by so called "Lebensschützern"). | "[...] in my practice I often have female patients in the consultation who have already been to other doctors and have not received the information there that I think would be adequate. For example, they were NOT ??? told that there were two different methods or that they were not informed about the deadlines and then somehow told that they still had plenty of time. Or that - yeah, actually that option [having an abortion] wasn't mentioned at all because immediately a maternity card [dt. "Mutterpass"] was handed over and [they were] congratulated and all the tests were done that are necessary in the first trimester without even asking if it's even a desired pregnancy." *W1G2TN07, Gynecologist* |
| **Collaboration as equal partners and involvement in decision-making** | Active involvement of and collaboration with the patient regarding decisions related to the patient’s health while taking into account the patient’s preference for involvement | deductive | - Sufficient background information was perceived as a basis for the decision-making process, often basic knowledge is available among women with UP, but this would not be sufficient to make a decision. - However, a prerequisite for making a decision is the availability of different methods. - According to participants, value-neutral information should be provided in order to enable women with UP to make their own decisions. | "So, in terms of shared decision making, the first thing that is really necessary is very often to first create a basis for the decision, so to speak. That is, to provide background information [...] because this is a decision that - there is no right or wrong in that sense. There is always only - how can I make a good decision for myself here?" *W2G1TN10, Counselor*  "And that's exactly the task, that we basically provide value-neutral education, information, whatever, so that the woman can make a decision herself." *W2G2TN17, Counselor* |
| **Involvement of family and friends** | Active involvement of and support for the patient’s relatives and friends to the degree that the patient prefers | deductive | - Support from family and friends was perceived as very helpful (e.g., that the partner is present during a medical abortion at home). - Participants citied that sometimes family had to be involved even if the person do not want them to be involved (e.g., children of women with UP have to translate during counseling or consultation with gynecologists because there are language barriers). | "Then I also always emphasize that it [the presence of another person] is not necessary for medical reasons, with the severity of the bleeding [during medical abortion in home-use], most women can manage that on their own, but it's often a farewell, exactly, and that's what we find - we always try to get women to organize that so that there's either the partner or at least another trusted person there." *W2G1TN12, Counselor* |
| Non-involvement of family and friends |  | inductive | - According to participants, the counselor or gynecologist should ask whether the non-involvement of other persons is desired by the women with UP. - The women with UP should be offered to speak alone with the counselor or gynecologist to have the opportunity to speak openly. | "In this case it is so that we actually, if it is several people or even if it is only the couple, that we often say in advance that we would also like to have a part of the conversation with the woman alone. [...] - it also happens from time to time that it comes out, okay, the woman actually doesn't want to be there right now and doesn't want an abortion at all." *W2G2TN17, Counselor* |
| **Empowerment of the person** | Recognition and active support of the patient’s ability and responsibility to self-manage his or her disease | deductive | - Participants reported that empowerment of women with UP is an aim of counseling and abortion care: > Women with UP should be encouraged to see and communicate what feels good for them. > Knowledge about medical background of pregnancy and abortion should be conveyed, body awareness and body knowledge should be strengthened. - Participants concluded that reservations among healthcare providers against abortion would prevent empowerment of women. | "[Counselors / physicians should convey in conversation,] That this is normal, to have feelings of conflict that actually seem to be in conflict, that at the same time you're relieved and yet you're terribly sad and just ashamed and so on and so forth." *W2G1TN13, Gynecologist* |
| Encouragement to make self-determined decisions |  | inductive | - Participants requested that women with UP should be encouraged in the decision-making process and the feeling that their decision will be the right one. - Women should be encouraged to consider their own life as valuable. - Sufficient background knowledge on the abortion procedure was perceived as important for a self-determined decision. | "I think it's important, above all, [...] to ensure care from beginning to end and to ensure company. And that for a self-determination of the woman or the girl who has to go the way there. So that women and girls are not patronized in their path of life, but that they learn to decide independently. In other words, empowerment. And not to deny women their own right to decide how to live their lives." *W1G2TN11, Counselor* |
| **Support of mental wellbeing** | Recognition of the patient’s emotional state and a set of behavior that ensures emotional support for the patient | deductive | - According to participants, psychological support should consist of: > an informative and structuring conversation, which can be reassuring in this emotionally situation  > providing time and space for the conversation and emotions of the women > addressing fears and providing security, being empathetic  > responding to individual needs of the women | "But for the women where it's like they're just very insecure and they're - and they feel torn and they're just plagued by all kinds of negative feelings because they're experiencing guilt, shame and all kinds of things and they're just generally unhappy - they're unhappy in those situations. That's where it's important to give that space." *W2G1TN13, Gynecologist* |
| Shame and guilt of women with UP |  | inductive | - According to participants, some women with UP are afraid of judgment are condemnation. - Shame was found particularly high in Muslim cultures. - Feelings of guilt and the associated desire to punish oneself would leads to a refusing of taking pain medication. - Participants observed that women with UP less likely talk to family and friends about the pregnancy and the abortion. - It was perceived as important to address possible feeling of shame and guilt in counseling and medical consultations. | "Well, I always provide pain medication and I tell the patients - they don't have to go through anything here, they don't have to endure anything here, they don't have to punish themselves, yeah. Sometimes you get the impression that women just don't want to take anything, according to the motto - I have to suffer it somehow. I have a certain guilt or something." *W2G2TN15, Gynecologist* |
| Addressing possible ambivalent feelings |  | inductive | - Participants underline that different and ambivalent emotions and feelings of the women can coexist in the context of abortion care. > Counselors / gynecologists should convey that it is normal to have ambivalent feelings regarding the abortion (e.g., being both relieved and sad after an abortion). | "That that's normal, to have feelings of conflict that actually seem to be in conflict, that at the same time you're relieved and yet you're terribly sad and just ashamed and so on and so forth." *W2G1TN13, Gynecologist* |
| **Support of physical wellbeing** | A set of behavior that ensures physical support for the patient (e.g. pain management, assistance with daily living needs) | deductive | No codes | No quotes |
| Pain management |  | inductive | - According to participants, taking pain medication should be discussed individually, as pain perception can vary between individuals. - Very often women would endure pain after an abortion because they want to punish themselves for having an abortion. | "[...] I tell the patients - they don't have to go through anything here, they don't have to endure anything here, and so it's like punishment, yes. Sometimes you get the impression that women just don't want to take anything, like according to the motto - I have to suffer it somehow. I have a certain guilt or something." *W2G2TN15, Gynecologist* |
| **Patient safety** | Treatment is planned to minimize the risk of negative consequences and errors in treatment. | deductive | - Participants highlighted the advantage of medical abortion: all medical examinations can be done without intervening in the body, which reduces risks. - Some participating gynecologists reported that they hand over their private cell phone number to women with UP so that they can reach a specialist directly in case of complications. | "Yes, that's the same with me. I mean, I give my private cell phone number then and it's extremely rare for a patient to call. But I think it gives her a sense of security. That she just knows, if something is wrong, I'll call the person who did it and who also has the professional competence." *W2G2TN15, Gynecologist* |
| Privacy protection |  | inductive | - According to participants, protecting the privacy of women with UP is essential and can be realized by  > separate rooms in the practice, especially for recovering after the abortion > enabling of confidentiality during the whole abortion process in the practice | "That you just have an environment, so to speak, where she is also withdrawn again. So she's not sitting in a waiting room in front of an audience [for recovering after the abortion], for example." *W2G2TN15, Gynecologist*   "And that is [...] confidentiality. I think it's striking that women often can't believe that [confidentiality will be provided]. Or question that very much, that it's really something that stays there." *W1G1TN03, Counselor* |
